# The Primary Resistance of *Helicobacter pylori* in Taiwan after the National Policy to Restrict Antibiotic Consumption and Its Relation to Virulence Factors—A Nationwide Study

**DOI:** 10.1371/journal.pone.0124199

**Published:** 2015-05-05

**Authors:** Jyh-Ming Liou, Chi-Yang Chang, Mei-Jyh Chen, Chieh-Chang Chen, Yu-Jen Fang, Ji-Yuh Lee, Jeng-Yih Wu, Jiing-Chyuan Luo, Tai-Cherng Liou, Wen-Hsiung Chang, Cheng-Hao Tseng, Chun-Ying Wu, Tsung-Hua Yang, Chun-Chao Chang, Hsiu‐Po Wang, Bor-Shyang Sheu, Jaw-Town Lin, Ming-Jong Bair, Ming-Shiang Wu, Taiwan Gastrointestinal Disease and Helicobacter Consortium

**Affiliations:** 1 Departments of Internal Medicine, National Taiwan University Hospital, National Taiwan University College of Medicine, Taipei, Taiwan; 2 Department of Internal Medicine, E- DA Hospital and I-Shou University, Kaohsiung County, Taiwan; 3 Departments of Internal Medicine, National Taiwan University Hospital, Yun-Lin Branch, National Taiwan University College of Medicine, Yun-Lin, Taiwan; 4 Department of Internal Medicine, Kaohsiung Municipal Ta-Tung Hospital, Kaohsiung Medical University, Kaohsiung, Taiwan; 5 Department of Medicine, National Yang-Ming University, School of Medicine, and Taipei Veterans General Hospital, Taipei, Taiwan; 6 Division of Gastroenterology, Department of Internal Medicine, Mackay Memorial Hospital, Taipei, Taiwan; 7 Division of Gastroenterology, Taichung Veterans General Hospital, Taichung, Taiwan, and Faculty of Medicine, School of Medicine, National Yang-Ming University, Taipei, Taiwan; 8 Department of Internal Medicine, Taipei Medical University Hospital, Taipei, Taiwan; 9 Institute of Clinical Medicine, National Cheng Kung University Hospital, College of Medicine, National Cheng Kung University, Tainan, Taiwan; 10 School of Medicine, Fu Jen Catholic University, New Taipei City, Taiwan; 11 Division of Gastroenterology, Department of Internal Medicine, Mackay Memorial Hospital, Taitung Branch, Taitung, Taiwan, and Department of Nursing, Meiho University, Pingtung, Taiwan; Kliniken der Stadt Köln gGmbH, GERMANY

## Abstract

**Objective:**

The Taiwan Government issued a policy to restrict antimicrobial usage since 2001. We aimed to assess the changes in the antibiotic consumption and the primary resistance of *H*. *pylori* after this policy and the impact of virulence factors on resistance.

**Methods:**

The defined daily dose (DDD) of antibiotics was analyzed using the Taiwan National Health Insurance (NHI) research database. *H*. *pylori* strains isolated from treatment naïve (N=1395) and failure from prior eradication therapies (N=360) from 9 hospitals between 2000 and 2012 were used for analysis. The minimum inhibitory concentration was determined by agar dilution test. Genotyping for CagA and VacA was determined by PCR method.

**Results:**

The DDD per 1000 persons per day of macrolides reduced from 1.12 in 1997 to 0.19 in 2008, whereas that of fluoroquinolones increased from 0.12 in 1997 to 0.35 in 2008. The primary resistance of amoxicillin, clarithromycin, metronidazole, and tetracycline remained as low as 2.2%, 7.9%, 23.7%, and 1.9% respectively. However, the primary levofloxacin resistance rose from 4.9% in 2000–2007 to 8.3% in 2008–2010 and 13.4% in 2011–2012 (p=0.001). The primary resistance of metronidazole was higher in females than males (33.1% vs. 18.8%, p<0.001), which was probably attributed to the higher consumption of nitroimidazole. Neither CagA nor VacA was associated with antibiotic resistance.

**Conclusions:**

The low primary clarithromycin and metronidazole resistance of *H*. *pylori* in Taiwan might be attributed to the reduced consumption of macrolides and nitroimidazole after the national policy to restrict antimicrobial usage. Yet, further strategies are needed to restrict the consumption of fluoroquinolones in the face of rising levofloxacin resistance.

## Introduction

The emergence of antibiotic resistance of *H*. *pylori* is a serious global problem, which is probably attributed to the increased consumption of antibiotics worldwide [1–4]. The outpatient antibiotic consumption in Taiwan increased since the initiation of National Health Insurance (NHI) in 1995. The prescription of antibiotics for upper respiratory tract infection was as high as 30% in 2000 [5,6]. Therefore, the Bureau of NHI of Taiwan issued a reimbursement regulation in 2001 to restrict the use of any antibiotics in patients with upper respiratory infection which was likely to be caused by virus [5,6]. The prescription of antibiotic would be reimbursed only in patients with evidence of bacterial infection, such as acute otitis media, sinusitis, etc [5,6]. The reimbursement of *H*. *pylori* eradication therapy was also limited to patients with peptic ulcer disease or mucosa-associated lymphoid tissue lymphoma. The most commonly used antibiotics in the first line treatment of *H*. *pylori* in Taiwan include amoxicillin, clarithromycin, or metronidazole. Bismuth quadruple therapy which includes tetracycline and metronidazole or levofloxacin triple therapy are the most commonly used regimens in the second line or third line therapies. Therefore, it is crucial to survey the changes of the consumptions of the above antibiotics and the changes in the prevalence of antibiotic resistance of *H*. *pylori* after the implementation of this national policy. Besides, it is also important to analyze the risk factors of antimicrobial resistance of *H*. *pylori* in order to further reduce the resistance rate. Several studies observed the lower eradication rate in patients with non-ulcer dyspepsia as compared to those with peptic ulcer disease, which linked to the hypothesis that there might been an association between the antibiotic resistance and the virulence factors of *H*. *pylori*, including the CagA and VacA genotypes [7–9]. However, the studies on this issue showed contradictory results.

Therefore, we organized a Taiwan Gastrointestinal Disease and *Helicobacter* Consortium [10,11] which consists of 9 hospitals in different geographic parts of Taiwan to assess the changes in the primary resistance of *H*. *pylori* and the consumption of antibiotics used for eradication therapies after the 2001 policy to restrict antimicrobial usage. The risk factors of antibiotic resistance, including the virulence factors (CagA and VacA genotypes) were also analyzed.

## Materials and Methods

### Patients and *H*. *pylori* Strains

This multicenter study was conducted in nine medical centers in Northern, Middle, Southern, and Eastern Taiwan between January 2001 and January 2012. *H*. *pylori* strains isolated from treatment naïve (N = 1395), failure from one (N = 192) and at least two (N = 158) eradication therapies between 2001 and 2012 were used for analysis. Demographic data, endoscopic diagnosis, and history of *H*. *pylori* eradication therapies were collected prospectively. The antibiotic resistance of strains collected before 2001 had been published and were not included in the present study to avoid duplication [12–14]. However, the reported prevalence of resistance before 2000 was used as historical control in order to assess the effectiveness of the national policy to restrict antibiotic usage [12–14]. The study protocol was approved by the Institutional Review Boards of National Taiwan University Hospital (NTUH). Written informed consents for the use of gastric biopsy specimens and *H*. *pylori* strains were obtained from all patients.

### Consumption of Antibiotics

The consumption of antibiotics was analyzed using the Longitudinal Health Insurance Database (LHID) of the National Health Insurance Database (NHIRD) which includes complete prescriptions of outpatient visits and hospital admissions and has a coverage rate greater than 99% of the total population (23 millions) in Taiwan [15]. The LHID contains randomly sampled 1,000,000 beneficiaries from the Registry for Beneficiary (ID) of the NHIRD [15]. The systemic random sample from 1997 to 2008 was representative of the whole population and contained all longitudinal reimbursement information [15]. The defined daily dose (DDD), the assumed average maintenance dose per day for a drug used as a main indication in adults, was calculated according to the definition of Drug Statistics Methodology of World Health Organization [16]. Subjects aged less than 18 years were excluded. Antibiotics administrated other than oral route were also excluded. We calculated the numbers of subjects aged 18 years or greater for each year from 1997 to 2008 as the denominators. The cumulative DDD (cDDD) of an antibiotic represented the total dose of that antibiotic prescribed during the study period. The DDD rate of the randomly sampled population was expressed as DDD per 1000 residents per day.

### Susceptibility test and genotyping

Genotyping and MICs were determined in NTUH. The minimum inhibitory concentrations (MICs) were determined by agar dilution tests using antibiotic-containing Mueller—Hinton agar supplemented with 5% defibrinated sheep blood. *H*. *pylori* ATCC 43504 was used as the quality control strain. The resistance breakpoints for amoxicillin, clarithromycin, levofloxacin, metronidazole, tetracycline, rifabutin, and rifampin were defined as greater than ≥0.5, ≥1, >1, ≥8, ≥0.5, ≥1, and >1mg/L, respectively [10,11,17]. The CagA gene and the VacA signal region (s1/2) and midregion (m1/2) mosaics were also determined by PCR method as described previously [11,17].

### Literature review of antibiotic resistance in Asia-Pacific regions

In order to compare the prevalence of antibiotic resistance of *H*. *pylori* in Taiwan to that in other countries in Asia-Pacific regions, we did a literature review (not a systematic review) to identify the most updated prevalence of primary “clarithromycin” and “metronidazole” resistance in Asia-Pacific regions. We searched the original full articles published in English after 2000 using the PubMed with the key words of “*H*. *pylori”*, “clarithromycin”, “metronidazole” and “resistance” in countries in Asia-Pacific regions. The search was limited to “title” and “abstracts”. Literatures that reported the secondary resistance were excluded. When there were two or more articles from a certain country, the most updated data were used to represent the resistance in that country.

### Statistical Analysis

Secondary resistance was defined as the resistance in patients who received at least one eradication therapy. Categorical data were compared using the chi-square test or Fisher’s exact test as appropriate. Continuous data were compared with Student’s t-test or ANOVA test and expressed as mean (SD). Logistic regression analyses were used to compute the odds ratios (ORs) and the 95% confidence interval (CI). All p-values were two-tailed, with the level of statistical significance specified as 0.05. The statistical analyses were performed using the SPSS 12.0.

## Results

### Prevalence of primary antimicrobial resistance

As shown in Table 1, the prevalence of primary resistance to clarithromycin, metronidazole, levofloxacin, amoxicillin, and tetracycline remained as low as 11.2% (95% CI 9.6%-13%), 25.7% (95% CI 23.5%-28.1%), 8.8% (95% CI 7.4%-10.4%), 2.3% (95% CI 1.7%-3.3%), and 2.7% (95% CI 2%-3.7%), respectively. The prevalence of primary resistance to rifabutin and rifampin remained as low as 2.2% (95% CI 1.3%-3.7%) and 14.3% (95% CI 12.4%-16.4%), respectively.

**Table 1:** Prevalence of primary and secondary antibiotic resistance of *Helicobacter pylori* in Taiwan.

	Primary Treatment-naive	Secondary Failed once	Secondary Failed at least twice	p-value
**Male**	51.6% (720/1395)	47.4% (101/192)	34.2% (54/158)	<0.001
**Age, mean (SD)**	51.8 (13.7)	52.3 (12.3)	52.6 (10.4)	0.852
**Clarithromycin**	11.2% (154/1378)	61.2% (115/188)	95.5% (149/156)	<0.001
**Levofloxacin**	8.8% (122/1384)	14.4% (27/188)	60.3% (94/156)	<0.001
**Metronidazole**	25.7% (355/1380)	33.5% (63/188)	64.7% (101/156)	<0.001
**Amoxicillin**	2.3% (32/1381)	3.7% (7/188)	8.3% (13/156)	<0.001
**Tetracycline**	2.7% (37/1353)	1.6% (3/188)	6.5% (10/155)	0.018
**Rifabutin**	2.2% (13/589)	2.8% (3/108)	1.0% (1/102)	0.643
**Rifampin**	14.3% (168/1177)	10.9% (15/138)	15% (21/140)	0.519
**Dual clarithromycin and metronidazole resistance**	4.3% (60/1383)	22.3% (42/188)	62.2% (97/156)	<0.001
**Multiple resistance (≥ 3 antibiotics)**	1.9% (26/1381)	7.4% (14/188)	49.3% (77/156)	<0.001

SD: standard deviation.

### Risk factors of resistance

History of eradication therapies was associated with increased risk of clarithromycin, levofloxacin, metronidazole, and amoxicillin resistance (Table 1). The secondary resistance of amoxicillin increased from 2.3% to 3.7% and 8.3% in patients who failed from one and at least two eradication therapies, respectively (p<0.001, Table 1). The primary levofloxacin resistance rose from 4.9% in 2000–2007 to 8.3% in 2008–2010 and 13.4% in 2011–2012 (p = 0.001) (Table 2). The primary resistance of levofloxacin was higher in older patients than in younger patients (Tables 2 and 3). The primary resistance of metronidazole was higher in women than in men (Tables 2 and 3). The primary resistance of metronidazole was lower in Eastern Taiwan (Table 3). Neither the CagA genotype nor VacA genotypes was associated clarithromycin, levofloxacin, metronidazole, amoxicillin, and tetracycline resistance (Table 2). The prevalence of the resistance of the five antibiotics was not different in patients with or without peptic ulcer disease, nor in patients with or without smoking.

**Table 2:** Primary antibiotic resistance of *Helicobacter pylori*—subgroup analysis.

	CLA	LEV	MET	AMO	TET
**Study periods**					
2000–2007	10.1% (31/306)	4.9% (15/306)	22.6% (69/305)	1.3% (4/305)	5.0% (15/298)
2008–2010	10.8% (78/721)	8.3% (60/726)	27.9% (202/723)	3.2% (23/724)	2.3% (16/703)
2011–2012	12.8% (45/351)	13.4% (47/352)	23.9% (84/352)	1.4% (5/352)	1.7% (6/352)
p-values	0.500	**0.001**	0.133	0.083	**0.019**
**Gender**					
Male	9.5% (67/702)	9.5% (67/705)	18.8% (132/703)	1.8% (13/704)	3.8% (26/689)
Female	13% (87/669)	8.2% (55/672)	33.1% (222/670)	2.8% (19/670)	1.7% (11/657)
p-values	0.043	0.389	**<0.001**	0.224	**0.019**
**Geographic areas**					
Northern	10.6% (69/653)	7.0% (46/655)	25.2% (165/654)	2.6% (17/653)	2.5% (16/640)
Middle	14.7% (47/319)	11.6% (37/320)	25.9% (83/320)	2.8% (9/320)	1.9% (6/320)
Southern	11.2% (32/286)	12.1% (35/289)	31.1% (89/286)	1.7% (5/288)	4.8% (13/273)
Eastern	5.0% (6/120)	3.3% (4/120)	15.0% (18/120)	0.8% (1/120)	1.7% (2/120)
p-values	**0.03**	**0.003**	**0.009**	0.536	0.124
**CagA genotype**					
Negative	9.5% (19/201)	9.4% (19/202)	30.2% (61/202)	1.5% (3/202)	0.5% (1/192)
Positive	11.5% (135/1175)	8.7% (103/1180)	25.0% (294/1176)	2.5% (29/1177)	3.1% (36/1159)
p-values	0.397	0.754	0.119	0.611	0.042
**VacA genotype**					
s1m1	10.9% (63/578)	7.6% (44/581)	26.9% (155/577)	2.4% (14/579)	4.3% (24/564)
s1m2	11.8% (76/643)	9.6% (62/646)	25.1% (162/646)	2.0% (13/645)	1.7% (11/634)
s2m1	0% (0/3)	0% (0/3)	66.7% (2/3)	0% (0/3)	0% (0/3)
s2m2	0% (0/5)	20% (1/5)	0% (0/5)	0% (0/5)	0% (0/5)
p-values	0.733	0.443	0.183	0.938	0.073

CLA: clarithromycin; LEV: levofloxacin; MET: metronidazole; AMO: amoxicillin; TET: tetracycline; GU: gastric ulcer; DU: duodenal ulcer; CagA: cytotoxin-associated gene A; VacA: Vacuolating cytotoxin A.

**Table 3:** Multivariate logistic analysis of risk factors for antimicrobial resistance.

	Odd Ratios (95% confidence interval)				
	CLA	LEV	MET	AMO	TET
Primary resistance					
**Age (years)**					
≥50 vs. <50	1.01 (0.71–1.44)	**1.57 (1.06–2.35)**	0.74 (0.58–0.95)	1.89 (0.86–4.12)	1.31 (0.64–2.7)
**Study periods**					
2008–2012 vs. 2000–2007	1.40 (0.84–2.33)	1.45 (0.77–2.74)	0.99 (0.69–1.43)	1.68 (0.5–5.6)	0.5 (0.22–1.15)
**Gender**					
Female vs. males	1.47 (1.04–2.08)	0.83 (0.57–1.22)	**2.13 (1.66–2.74)**	1.55 (0.76–3.16)	0.46 (0.22–0.95)
**Underlying disease**					
Gastric cancer vs. others	3.9 (2.24–6.8)	0.45 (0.16–1.31)	1.11 (0.66–1.86)	1.01 (0.22–4.79)	3.34 (1.35–8.26)
**Geographic areas**					
Eastern vs. others	0.58 (0.23–1.44)	0.52 (0.17–1.58)	**0.49 (0.27–0.90)**	0.56 (0.06–5.0)	0.48 (0.1–2.22)

CLA: clarithromycin; LEV: levofloxacin; MET: metronidazole; AMO: amoxicillin; TET: tetracycline.

### The trend of resistance in relation to the consumption of antibiotics

**Fig 1:**
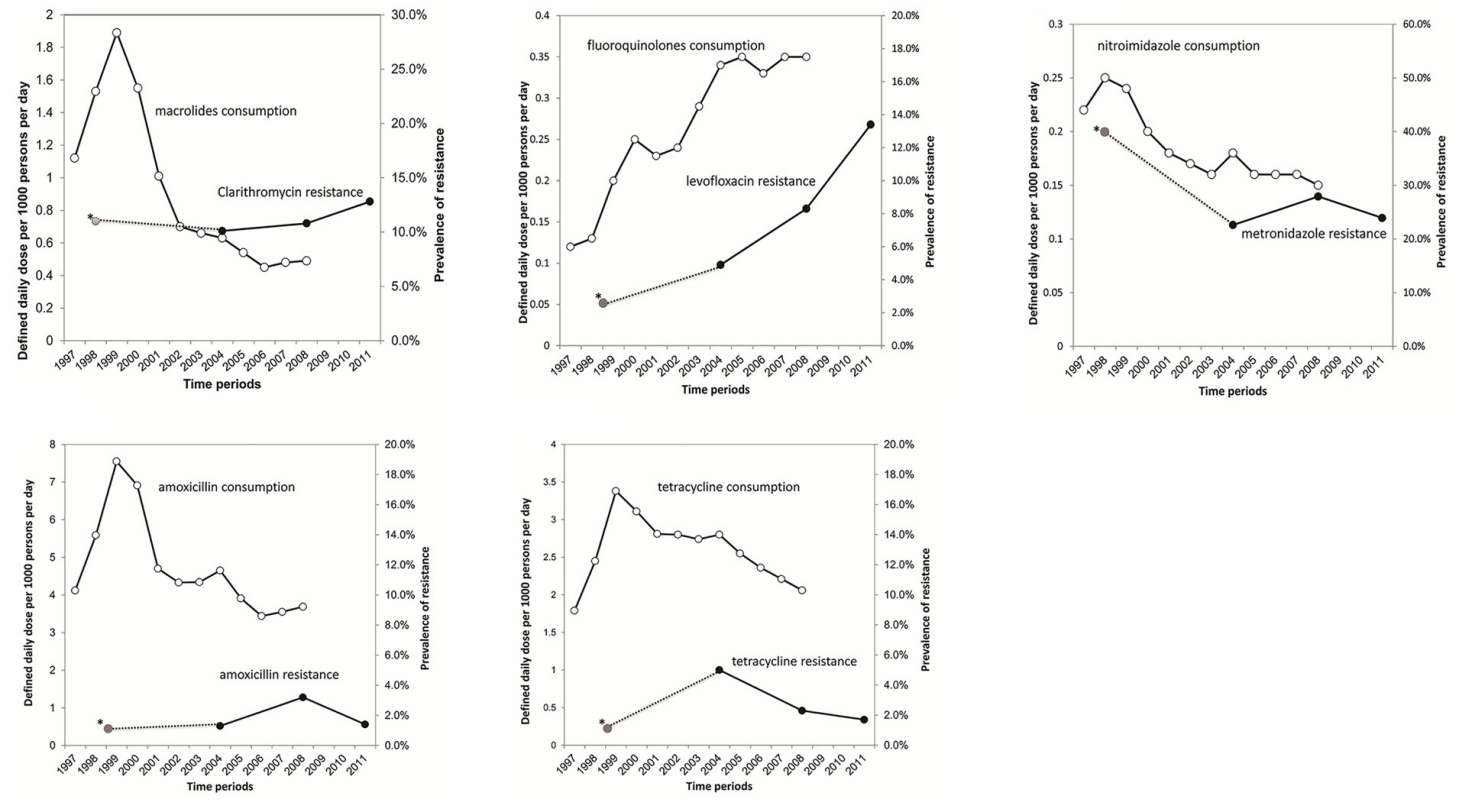
The time trend of outpatient antibiotic consumption and primary resistance of *Helicobacter pylori* in Taiwan. We showed trends of reduced consumption of macrolides, nitroimidazole, amoxicillin, and tetracycline after the national policy to restrict antimicrobial usage. The primary resistance of clarithromycin, metronidazole, amoxicillin, and tetracycline remained low and the trends were stable during 2001–2011. In contrast, we observed a trend of increased consumption of fluoroquinolones and a trend of rising primary levofloxacin resistance despite the national policy to restrict antimicrobial usage. *The antibiotic resistance of strains collected before 2001 had been published and were not included in the present study to avoid duplication [13–15]. The antibiotic resistances before 2001 were presented in dotted lines in Fig 1 to better demonstrate the trends of resistance before and after the policy to restrict antimicrobial usage [13–15].

We observed trends of reduced consumption of clarithromycin, metronidazole, amoxicillin, and tetracycline after the 2001 policy to restrict antimicrobial usage, except for fluoroquinolone (Fig 1 and S1 Table). The DDD rate of amoxicillin, tetracycline, macrolides, and nitroimidazole reduced from 6.91, 3.11, 1.55, and 0.20 DDD per 1000 persons per day in 2000 before the national policy to restrict antibiotic usage to 3.69, 2.06, 0.49, and 0.15 DDD per 1000 persons per day in 2008, respectively (Fig 1 and S1 Table). The primary resistance rate of clarithromycin, metronidazole, and amoxicillin remained stable during 2001 and 2012 (Fig 1). The antibiotic resistances before 2001 were presented in dotted lines in Fig 1 to better demonstrate the changes in the trends of resistance before and after the policy to restrict antimicrobial usage [13–15]. However, the DDD rate of fluoroquinolones increased from 0.12, 0.25, 0.34, and 0.35 DDD per 1000 persons per day in 1997, 2000, 2004, and 2008, respectively (Fig 1B). The increased fluoroquinolones consumption appeared to correlate with the rising primary resistance rate of levofloxacin (Fig 1B). The DDD rate of nitroimidazole was higher in females than in males (S1 Fig).

### The prevalence of primary resistance in Taiwan and other countries in Asia-Pacific regions

**Fig 2:**
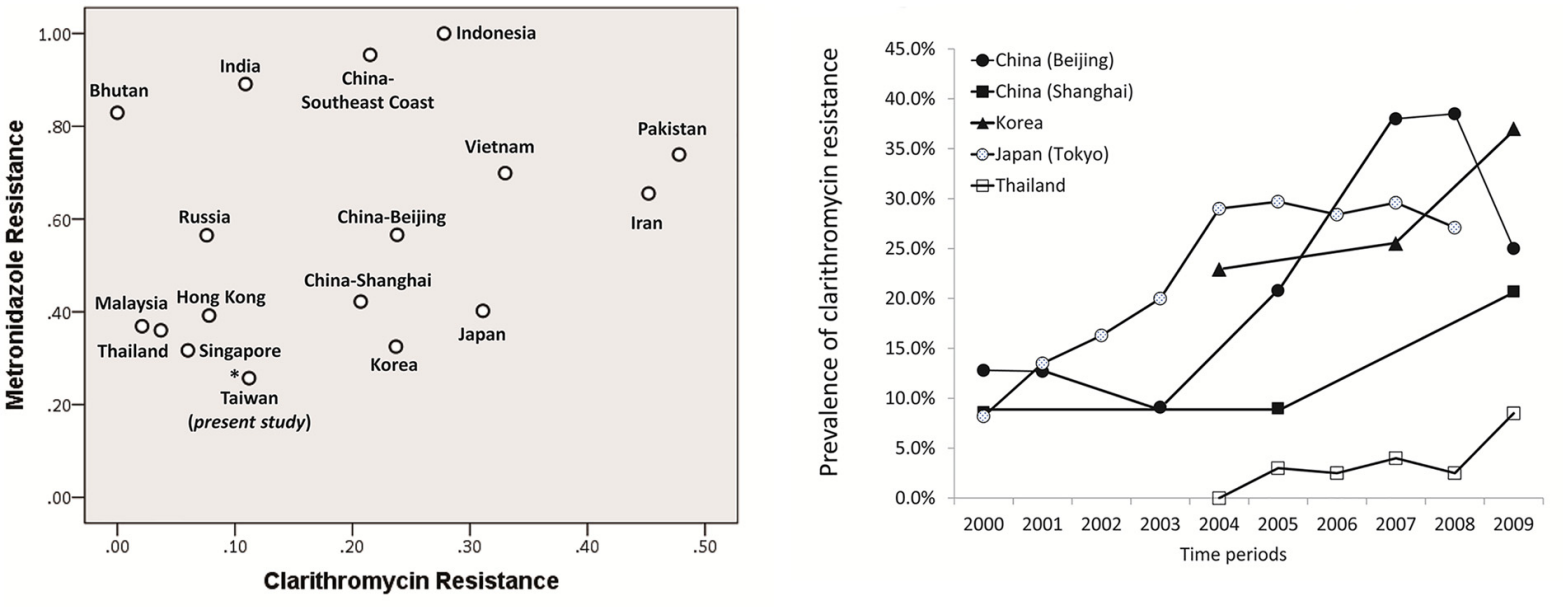
The prevalence of primary clarithromycin and metronidazole resistance of *Helicobacter pylori* in Asia-Pacific regions. Fig 2A showed that the prevalence of primary clarithromycin and metronidazole resistance remained low in Taiwan, as compared to many other countries in Asia-Pacific regions ([18–25] and S2 Table). Fig 2B showed the trend of clarithromycin resistance in Asia-Pacific regions [12, 18–21].

The updated primary resistant rate in Asia-Pacific region was shown in Fig 2 and S2 Table [18–25]. The results from the present study showed that the primary resistant rates of clarithromycin and metronidazole remained low in Taiwan, as compared to many other countries in this region (Fig 2A and S2 Table) [18–25]. The trend of primary resistance of clarithromycin remained stable during 2001 and 2012 in Taiwan, compared to the rising resistance during this time period in China, Japan, and Korea (Fig 2B and S2 Table).

## Discussion

In this multicenter surveillance program, we found that the primary resistance rate of clarithromycin, metronidazole, amoxicillin, and tetracycline remained low in Taiwan, which might be attributed to the reduced consumption of these antibiotics in Taiwan (Figs 1 and 2A). Compared to the rising resistance during this time period in China, Japan, and Korea (Fig 2B and S2 Table) [20–24], the trend of primary resistance of clarithromycin remained stable during 2001 and 2011 in Taiwan, which could be attributed to the 2001 national policy to restrict the usage of these antibiotics. However, we observed a rising prevalence of primary levofloxacin resistance which might be attributed to the increased consumption of fluoroquinolones despite this policy (Fig 1B). This indicated that further strategies are warranted to restrict the consumption of fluoroquinolones.

The time trend of clarithromycin resistance of *H*. *pylori* in relation to macrolides consumption has been reported in some studies. In Japan, the primary resistance increased from 4% to 6% and 15% in 1996, 1998, and 2000, respectively, which correlated with the increase in total amount of macrolides consumption [26]. The primary clarithromycin resistance remained as low as 1.1–3.3% in Lithuania during 2003–2007 and 0–3.3% in Estonia during 1996–2000 [27,28]. The low resistance rate might be attributed to the low consumption of macrolides in Lithuania (DDD rate ranging from 1.2 to 1.8) and Estonia (DDD rate ranging from 1 to 1.5) [27,28]. In Finland, they observed a trend of increase in clarithromycin resistance between 2000 and 2006, but a trend of reduction between 2006 and 2008 [29]. However, the changes in the clarithromycin resistance did not correlate with the changes in macrolides consumption in Finland [29]. In Belgium, the primary clarithromycin resistance increased from 5.2% in 1995 to 15.3% in 2004, but reduced to 10.5% in 2009, which was probably attributed to the reduced consumption of macrolides [30]. In a recent multi-center study in Europe, Megraud and colleagues further demonstrated that the primary resistance of clarithromycin in a country correlated with the amount of clarithromycin consumption in that country [1]. However, none of the previous studies assessed the time trends between levofloxacin resistance and fluoroquinolones consumption.

In the present study, we found that whereas the consumption of macrolides, amoxicillin, nitroimidazole, and tetracycline reduced after the national policy to restrict antibiotic usage since 2001, the consumption of fluoroquinolones increased. This might be the explanation for the rising primary levofloxacin resistance. Besides, the secondary resistance of levofloxacin also increased to 65% in strains isolated from patients who failed at least two eradication therapies. The rising resistance rate was probably attributed to the increasing use of levofloxacin as the second line therapy in Taiwan [10,11]. A similar trend of high secondary resistance to fluoroquinolones of *H*. *pylori* has been reported in Germany [31]. The increased consumption of fluoroquinolones might be attributed to the recommendations from treatment guidelines that fluoroquinolone monotherapy may be used as alternative therapy to beta-lactam-macrolide combination therapy in the first line treatment of community acquired pneumonia (CAP) [32]. Another explanation for the increased consumption of fluoroquinolones was that the use of fluoroquinolones in urinary tract infection was not restricted in the national policy. Therefore, a more judicious use of fluoroquinolone is important to prevent the emergency of fluoroquinolone resistant *H*. *pylori* strains. We proposed that fluoroquinolones should be reserved as rescue therapy for *H*. *pylori* infection or susceptibility testing before first line therapy shows that the strain is resistant to other antibiotics. Whether the fluoroquinolones should be reserved as second line therapy in CAP and urinary tract infection is also an important issue to be addressed in future studies and treatment guidelines.

The strength of our study included the large sample size (N = 1745), the multicenter study design which is representative of Taiwan, and the use of the Taiwan NHIRD to obtain the amount of antibiotic consumption. Because we conducted a nationwide survey in both symptomatic and asymptomatic subjects, the positive rate of CagA (85%) was relatively lower compared to that reported from other East Asian countries [33].Nevertheless, there were some limitations with this study. The major limitation was that the strains collected before 2000 were not included in this study because the data had been published previously [12–14]. Previous studies from Taiwan showed that the prevalence of clarithromycin and metronidazole resistance was 11% and 41%, respectively, before 2000 [12–14]. The prevalence of levofloxacin was as low as 2.8% during 1998 and 2003. Therefore, we cited the published data in this study further demonstrate the changes in the trends of antibiotic resistance before and after the policy to restrict antibiotic usage. Since the change of antibiotic consumption is expected to precede the changes in the prevalence of resistance, we also included the data of antibiotic consumption between 1997 and 2000. Secondly, there are other factors that might affect the consumption of antibiotics in addition to the policy to restrict antibiotic usage, such as the treatment consensus or guidelines. The causal relationship for the changes in the consumption of antibiotics and the policy to restrict antibiotic usage could not be established by the serial cross-sectional surveys. Yet, it is difficult to conduct randomized trial at the national level to prove the efficacy of this policy on the consumption of antibiotics and the prevalence of resistance. Thirdly, the underlying mechanisms for the higher clarithromycin and tetracycline resistance in patients with gastric cancer and the higher clarithromycin resistance in females need to be clarified and validated in future studies. Fourthly, we performed a random sampling of 1 million outpatients out of the 23 million people in Taiwan to analyze the antibiotic consumption because of the difficulty in obtaining the entire database from the Bureau of NHI. Nevertheless, the sampling was random and selection bias is less likely. Finally, the MICs f rifabutin, rifampin, and tetracycline were not determined in all strains because the prevalence of resistance were relative lower. The determination of MIC failed in some strains. Nevertheless, the sample size of this study was large and the failure rate was less than 1.5%.

In conclusion, the reduced consumption of antibiotics after the policy to restrict antibiotic usage might contribute to the low primary resistance of *H*. *pylori* in Taiwan. The rising prevalence of levofloxacin resistance might be attributed to the increased consumption of fluoroquinolones in Taiwan and more judicious use of fluoroquinolones is mandatory in future clinical practice. The virulence genotypes of *H*. *pylori*, the CagA and VacA, are not associated with antibiotic resistance. Continuous surveillance of antimicrobial resistance and the judicious use of antibiotic are important strategies to prevent the emergence of resistance.

## Supporting Information

S1 Fig Consumption of nitroimidazole according to gender according to the Taiwan National Health Insurance research database. (TIF)

S1 Table The defined daily dose (DDD) of antibiotic use in Taiwan between 1997 and 2008. (DOCX)

S2 Table The updated prevalence of antimicrobial resistance in Asia-Pacific Regions. (DOCX)
