# Supplementary figures and images for: The Primary Resistance of Helicobacter pylori in Taiwan after the National Policy to Restrict Antibiotic Consumption and Its Relation to Virulence Factors—A Nationwide Study
Source: PLoS One. 2015 May 5;10(5):e0124199. doi: 10.1371/journal.pone.0124199 (PMC4420283; doi:10.1371/journal.pone.0124199)

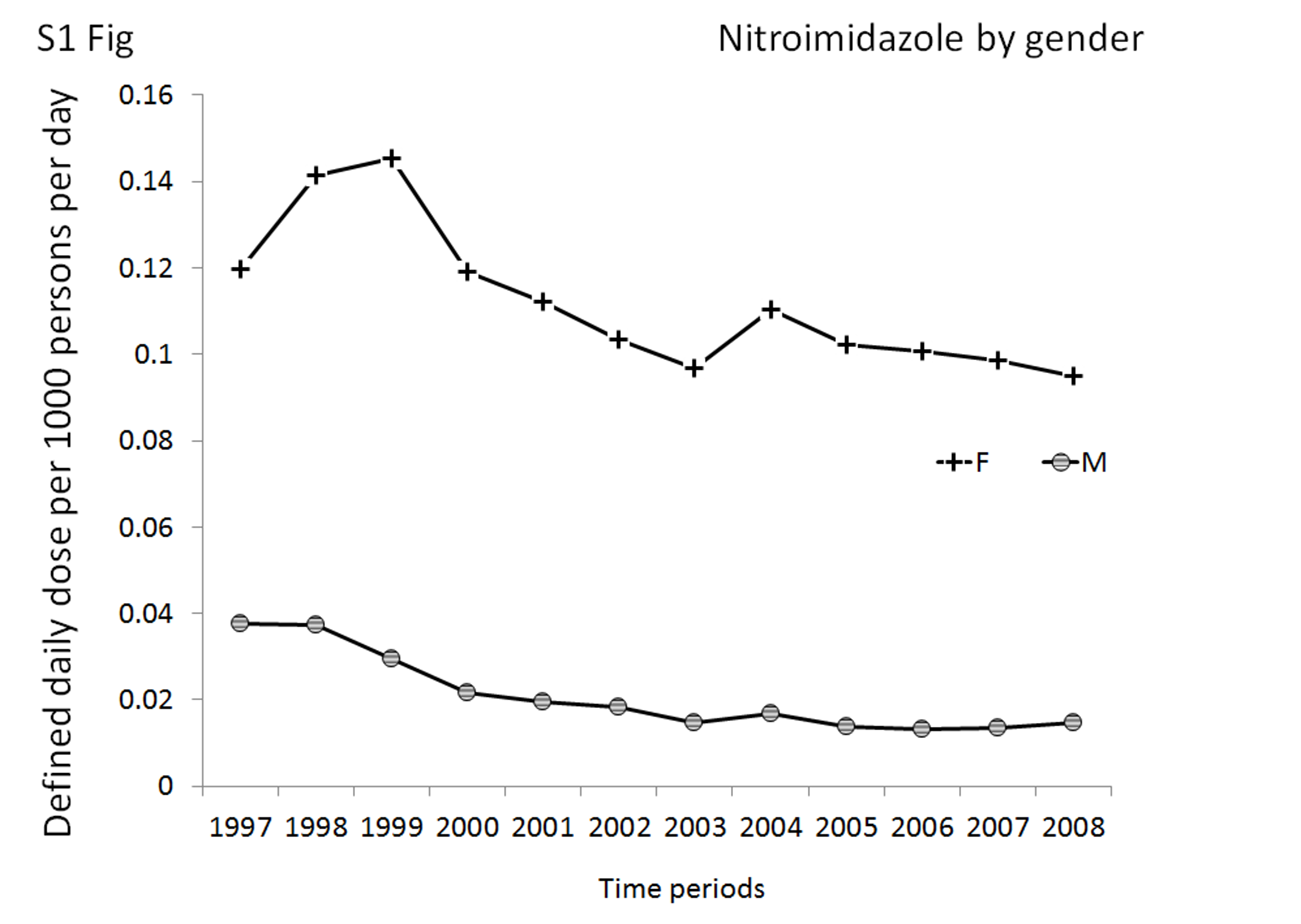

Supplement: S1 Fig — (TIF) [file pone.0124199.s001.tif]
