# Supplementary material for: The Primary Resistance of Helicobacter pylori in Taiwan after the National Policy to Restrict Antibiotic Consumption and Its Relation to Virulence Factors—A Nationwide Study
Source: PLoS One. 2015 May 5;10(5):e0124199. doi: 10.1371/journal.pone.0124199 (PMC4420283; doi:10.1371/journal.pone.0124199)
Supplement: S1 Table — (DOCX) [file pone.0124199.s002.docx]

**S1 Table. The defined daily dose (DDD) of antibiotic use in Taiwan between 1997 and 2008**

| **year** | **No of persons** | **Consumption of antibiotics** | | | | | | | | | |
| --- | --- | --- | --- | --- | --- | --- | --- | --- | --- | --- | --- |
|  |  | **Amoxicillin** | | **Tetracycline** | | **Macrolides** | | **Nitroimidazole** | | **Fluoroquinolone** | |
|  |  | cDDD | DDD rate | cDDD | DDD rate | cDDD | DDD rate | cDDD | DDD rate | cDDD | DDD rate |
| 1997 | 633110 | 951814.5 | 4.12 | 414425.8 | 1.79 | 258267.3 | 1.12 | 50041.3 | 0.22 | 27323.6 | 0.12 |
| 1998 | 651617 | 1330188 | 5.59 | 581626 | 2.45 | 363253.9 | 1.53 | 58695.7 | 0.25 | 31849.1 | 0.13 |
| 1999 | 669337 | 1845641 | 7.55 | 825185.4 | 3.38 | 462795.7 | 1.89 | 59059.2 | 0.24 | 48626.3 | 0.20 |
| 2000 | 686561 | 1730658 | 6.91 | 779949 | 3.11 | 387974.3 | 1.55 | 48872.7 | 0.20 | 61976.3 | 0.25 |
| 2001 | 703034 | 1205318 | 4.70 | 721196.8 | 2.81 | 258448.3 | 1.01 | 46973.6 | 0.18 | 59382.8 | 0.23 |
| 2002 | 718688 | 1136241 | 4.33 | 735065.1 | 2.80 | 182495.9 | 0.70 | 44454.9 | 0.17 | 61842.1 | 0.24 |
| 2003 | 733463 | 1161285 | 4.34 | 734224.7 | 2.74 | 176645.8 | 0.66 | 41596.9 | 0.16 | 76834.3 | 0.29 |
| 2004 | 746983 | 1267429 | 4.65 | 763610.1 | 2.80 | 171109.7 | 0.63 | 48387.8 | 0.18 | 93430.6 | 0.34 |
| 2005 | 759172 | 1083159 | 3.91 | 707162.8 | 2.55 | 148766.4 | 0.54 | 44911.5 | 0.16 | 98264.6 | 0.35 |
| 2006 | 772706 | 970318 | 3.44 | 666636.1 | 2.36 | 127126.3 | 0.45 | 44832.1 | 0.16 | 93539.6 | 0.33 |
| 2007 | 787025 | 1018962 | 3.55 | 633860.4 | 2.21 | 138677.3 | 0.48 | 44922.8 | 0.16 | 99456.2 | 0.35 |
| 2008 | 800372 | 1078664 | 3.69 | 601228.9 | 2.06 | 142842.4 | 0.49 | 44612.5 | 0.15 | 102781.2 | 0.35 |

cDDD: cumulative defined daily dose; DDD rate: defined daily dose per 1000 persons per day.
