# Supplementary material for: The Primary Resistance of Helicobacter pylori in Taiwan after the National Policy to Restrict Antibiotic Consumption and Its Relation to Virulence Factors—A Nationwide Study
Source: PLoS One. 2015 May 5;10(5):e0124199. doi: 10.1371/journal.pone.0124199 (PMC4420283; doi:10.1371/journal.pone.0124199)
Supplement: S2 Table — (DOCX) [file pone.0124199.s003.docx]

**S1 Table. The updated prevalence of antimicrobial resistance in Asia-Pacific Regions**

| **Country** | **Region** | **Authors** | **Ref** | **Year** | **Method** | **Clarithromycin** | **Metronidazole** | **Levofloxacin** | **amoxicillin** | **Tetracycline** |
| --- | --- | --- | --- | --- | --- | --- | --- | --- | --- | --- |
| **Bhutan** | Thimpu | Vilaichone et al. | 1 | 2010 | E-test | 0 | 82.9%  (92/111) | 2.7% (3/111) | 0 | 0 |
| **China** | Southeast Coastal | Su et al. | 2 | 2010-2012 | agar dilution | 21.5% (3810/17731) | 95.4% (16908/17731) | 20.6% (3661/17731) | 0.1% (22/17731) | - |
|  | Beijing | Gao et al. | 3 | 2000-2009 | E-test | 23.8% (69/290) | 56.6% (164/290) | 36.9% (38/103) | 0.3% (1/290) | 1% (1/104) |
|  | Shanghai | Sun et al. | 4 | 2009 | Agar dilution | 20.7% (28/135) | 42.2% (57/135) | 32.6% (44/135) | 0 | 0.7% (1/135) |
| **Hong Kong** | Hong Kong | Gu et al | 5 | 2004 | E-test | 7.8% (8/102) | 39.2% (40/102) | - | 0% | - |
| **India** | North | Singh et al | 6 | 2005-2006 | Agar dilution | 4.8% (3/63) | 100% (63/63) | - | 65% (41/63) | - |
|  | Chennai | Dharmalingam et al. | 7 | before 2003 | E-test | 10.9% (12/110) | 89.1% (98/110) | 0.9%*(1/110) | 0.9% (1/110) | - |
|  | Chennai | Thyagarajan et al. | 8 | before 2003 | E-test | 44.7% (116/259) | 77.9% (202/259) | 2.7%* (7/259) | 32.8% (85/259) | 1.54% (4/259) |
| **Indonesia** | Jakarta | Kumala et al. | 9 | before 2006 | disc diffusion | 27.8% (20/72) | 100% (72/72) | 1.4% (1/72) | 19.4% (14/72) | - |
| **Iran** | Tehran | [Abadi](http://www.ncbi.nlm.nih.gov/pubmed?term=Abadi%20AT%5BAuthor%5D&cauthor=true&cauthor_uid=22203563) et al. | 10 | 2009 | disc diffusion | 45.2% (89/197) | 65.5% (129/197) | 34.5%* (68/197) | 23.9% (47/197) | 37.1% (73/197) |
| **Japan** | Matsumoto | Okamura et al. | 11 | 2000-2013 | Microbroth dilution | 31.1% (334 /1073). | 40.2% (431 of 1073). | - | - | - |
| **Korea** | Gyeonggi-do | Lee et al | 12 | 2009-2012 | Agar dilution | 23.7%  (27/114) | 32.5%  (37/114) | 28.1%  (32/114) | 14.9%  (17/114) | 31%  (35/114) |
| **Malaysia** | Kuala Lumpur | [Ahmad](http://www.ncbi.nlm.nih.gov/pubmed?term=Ahmad%20N%5BAuthor%5D&cauthor=true&cauthor_uid=21241412) et al. | 13 | 2004-2007 | E-test | 2.1% (4/187) | 36.9% (69/187) | 1.1% (2/187) | 0% | 0% |
| **Pakistan** | Islamabad | Rasheed et al. | 14 | 2011-2012 | E-test | 47.8% (22/46) | 73.9% (34/46) | 13%* (6/46) | 54.3% (25/46) | 4.3% (2/46) |
| **Thailand** | Nationwide | Vilaichone et al. | 15 | 2004-2012 | E-test | 3.7% (15/400) | 36% (144/400) | 7.2% (15/208) | 5.2% (21/400) | 1.7% (7/400) |
| **Vietnam** | Ho Chi Minh and Hanoi | Binh et al. | 16 | 2008 | E-test | 33.0% (34/103) | 69.9% (72/103) | 18.4% (19/103) | 0% | 5.8% (6/103), |
| **Taiwan** | **Nationwide** | **Present study** |  | **2001-2012** | **Agar dilution** | **11.2% (154/1378)** | **25.7% (355/1380)** | **8.8% (122/1384)** | **2.3% (32/1381)** | **2.7% (37/1353)** |

**References**

1. Vilaichone RK, Yamaoka Y, Shiota S, Ratanachu-ek T, Tshering L, et al. (2013) Antibiotics resistance rate of *Helicobacter pylori* in Bhutan. World J Gastroenterol19:5508-5512.
2. Su P, Li Y, Li H, Zhang J, Zhang J, Lin L, et al. (2013) Antibiotic resistance of *Helicobacter pylori* isolated in the Southeast Coastal Region of China. Helicobacter 18:274-279.
3. Gao W, Cheng H, Hu F, Li J, Wang L, et al. (2010) The evolution of *Helicobacter pylori* antibiotics resistance over 10 years in Beijing, China. Helicobacter 15:460-466.
4. Sun QJ, Liang X, Zheng Q, Gu WQ, Liu WZ, et al. (2010) Resistance of *Helicobacter pylori* to antibiotics from 2000 to 2009 in Shanghai. World J Gastroenterol;16:5118-5121.
5. Gu Q, Xia HH, Wang JD, Wong WM, Chan AO, et al. (2006) Update on clarithromycin resistance in *Helicobacter pylori* in Hong Kong and its effect on clarithromycin-based triple therapy. Digestion 273:101-106.
6. Singh V, Mishra S, Maurya P, Rao G, Jain AK,et al. (2009) Drug resistance pattern and clonality in *H. pylori* strains. J Infect Dev Ctries 3:130-136.
7. Dharmalingam S, Rao UA, Jayaraman G, Thyagarajan SP. (2003) Relationship of plasmid profile with the antibiotic sensitivity pattern of *Helicobacter pylori* isolates from peptic ulcer disease patients in Chennai. Indian J Med Microbiol 21:257-261.
8. [Thyagarajan SP](http://www.ncbi.nlm.nih.gov/pubmed?term=Thyagarajan%20SP%5BAuthor%5D&cauthor=true&cauthor_uid=14675265), [Ray P](http://www.ncbi.nlm.nih.gov/pubmed?term=Ray%20P%5BAuthor%5D&cauthor=true&cauthor_uid=14675265), [Das BK](http://www.ncbi.nlm.nih.gov/pubmed?term=Das%20BK%5BAuthor%5D&cauthor=true&cauthor_uid=14675265), Ayyagari A, Khan AA, et al. (2003) Geographical difference in antimicrobial resistance pattern of *Helicobacter pylori* clinical isolates from Indian patients: Multicentric study. [J Gastroenterol Hepatol](http://www.ncbi.nlm.nih.gov/pubmed/?term=J+Gastroenterol+Hepatol.+2003%3B18%3A1373-8##) 18:1373-1378.
9. [Kumala W](http://www.ncbi.nlm.nih.gov/pubmed?term=Kumala%20W%5BAuthor%5D&cauthor=true&cauthor_uid=17333742), [Rani A](http://www.ncbi.nlm.nih.gov/pubmed?term=Rani%20A%5BAuthor%5D&cauthor=true&cauthor_uid=17333742). (2006) Patterns of *Helicobacter pylori* isolate resistance to fluoroquinolones, amoxicillin, clarithromycin and metronidazoles. [Southeast Asian J Trop Med Public Health](http://www.ncbi.nlm.nih.gov/pubmed/?term=Southeast+Asian+J+Trop+Med+Public+Health.+2006%3B37%3A970-4##) 37:970-974.
10. [Abadi AT](http://www.ncbi.nlm.nih.gov/pubmed?term=Abadi%20AT%5BAuthor%5D&cauthor=true&cauthor_uid=22203563), [Taghvaei T](http://www.ncbi.nlm.nih.gov/pubmed?term=Taghvaei%20T%5BAuthor%5D&cauthor=true&cauthor_uid=22203563), [Mobarez AM](http://www.ncbi.nlm.nih.gov/pubmed?term=Mobarez%20AM%5BAuthor%5D&cauthor=true&cauthor_uid=22203563), , Carpenter BM, Merrell DS. (2011) Frequency of antibiotic resistance in *Helicobacter pylori* strains isolated from the northern population of Iran. [J Microbiol](http://www.ncbi.nlm.nih.gov/pubmed/?term=J+Microbiol+2011%3B49%3A987-93##) 49:987-993.
11. Okamura T, Suga T, Nagaya T, Arakura N, Matsumoto T, et al. (2014) Antimicrobial resistance and characteristics of eradication therapy of *Helicobacter pylori* in Japan: a multi-generational comparison. [Helicobacter](http://www.ncbi.nlm.nih.gov/pubmed/24758533) 19:214-220.
12. Lee JW, Kim N, Kim JM, Nam RH, Chang H, et al. (2013) Prevalence of primary and secondary antimicrobial resistance of *Helicobacter pylori* in Korea from 2003 through 2012. Helicobacter 18:206-214.
13. [Ahmad N](http://www.ncbi.nlm.nih.gov/pubmed?term=Ahmad%20N%5BAuthor%5D&cauthor=true&cauthor_uid=21241412), [Zakaria WR](http://www.ncbi.nlm.nih.gov/pubmed?term=Zakaria%20WR%5BAuthor%5D&cauthor=true&cauthor_uid=21241412), [Mohamed R](http://www.ncbi.nlm.nih.gov/pubmed?term=Mohamed%20R%5BAuthor%5D&cauthor=true&cauthor_uid=21241412). (2011) Analysis of antibiotic susceptibility patterns of Helicobacter pylori isolates from Malaysia. [Helicobacter](http://www.ncbi.nlm.nih.gov/pubmed/?term=Helicobacter.+2011%3B16%3A47-51##) 16:47-51.
14. Rasheed F, Campbell BJ, Alfizah H, Varro A, Zahra R, et al. (2014) Analysis of Clinical Isolates of *Helicobacter pylori* in Pakistan Reveals High Degrees of Pathogenicity and High Frequencies of Antibiotic Resistance. Helicobacter 19:387-399.
15. Vilaichone RK, Gumnarai P, Ratanachu-Ek T, Mahachai V. (2013) Nationwide survey of *Helicobacter pylori* antibiotic resistance in Thailand. Diagn Microbiol Infect Dis 77:346-349.
16. Binh TT, Shiota S, Nguyen LT, Ho DD, Hoang HH, et al. (2013) The incidence of primary antibiotic resistance of *Helicobacter pylori* in Vietnam. J Clin Gastroenterol 47:233-238.
